# Selections and Abstracts

**Published:** 1900-04

**Authors:** 


### SELECTIONS AND ABSTRACTS.

### Four Cases of Diabetes Mellitus of Apparent Bacterial Origin, and Their Successful Treatment.

By J. P. Sheridan, M.D.

In the latter part of 1898 a writer in the *Medical Record* related his experience with bichloride of mercury in the treatment of diabetes mellitus and advanced the novel theory of the bacterial origin of this affection.

At the time of publication of the article in question, I had some diabetics under treatment. As a moderately rigid antidiabetic diet and the time-honored remedies did not check the glycosuria in my patients, I adopted the newly proffered theory and eagerly prescribed the advocated chemical.

To-day, after a year's trial of germicidal remedies in diabetes, I have become a firm believer in the bacterial origin of diabetes. It is true, the bichloride of mercury did not prove a success in my hands, but this only tends to demonstrate the existence of a peculiar diabetic toxine, which has to be combated by other means.

Bichloride of mercury and auri et sodii chloridum, which latter is so much lauded of late by a Chicago physician, possess some of these desiderata, but neither proved of any success in my hands in the treatment of diabetes mellitus. This non-success is due to three factors:

A. The specific toxine of diabetes is affected only by a specific antiseptic.

B. Bichloride of mercury or auri et sodii chloridum, when pushed to their physiological tolerance, do not effect the decline of the glycosuria.

C. Bichloride of mercury as well as chloride of gold and sodium, when administered for any length of time and in larger doses, reduce the oxidizing power of the red-blood cells, thereby weakening the system and producing rapid emaciation.

The remedy answering all the demands for an ideal antidiabetic

I find in a combination of bromide of gold with bromide of arsenic, called by its makers, "arsenauro." This preparation undoubtedly exerts a specific influence upon the bacteria and the toxine of diabetes mellitus, which is elucidated by the following case:

CASE I. Mr. C. L., aged fifty, American, clerk, consulted me on June 8, 1898. Family history was negative. Patient complained of polyuria, the existence of which dated back about three months. The frequency in urination he thought to be due to a stricture, the possible result of a neglected gonorrhea. Patient had a moderate appetite, felt quite thirsty at times, and had lost some weight. The urine (which was voided to the amount of about seven pints daily) on June 10, 1898, showed a specific gravity of 1.038 and contained 7.1 per cent. of sugar, as ascertained by means of Stern's urinoglucosometer. A restricted diet and the administration of codeine caused only a moderate improvement of the symptoms. Bichloride of mercury, which was given for the last three weeks of December, 1898, in the doses recommended, not only produced no beneficial influence whatsoever upon the diabetic condition, but actually aggravated the condition of the patient. Early in July, 1898, my attention was drawn to the chloride of gold and sodium, which was handed to the patient in tablet form and administered first in doses of a fiftieth of a grain. The dose was gradually increased to a twentieth of a grain. After five weeks' trial of this drug it had to be abandoned, as the condition of the patient had become alarming in the meantime.

At about this period I ran across an article in the *New York Medical Journal* regarding the use of arsenauro in diabetes and determined to test this product, having previously used it with satisfactory results in malarial toxemia.

On February 7, 1899, eight drops of arsenauro were given in half a glass of water three times daily. The restricted diet was ordered to be continued. Patient reported to me in one week. The glycosuria and polyuria were greatly diminished. The feeling of thirst was not experienced any longer, and he expressed himself as feeling perfectly well. The dose of arsenauro was gradually increased until he reached his full limit of toleration, which supervened at fifty drops. The quantity was lessened to forty-five drops, and continued in this dose for sixteen weeks. After this period I examined the urine, which revealed a specific gravity of 1.020 and was absolutely free of sugar. Patient was discharged as cured, with the instruction to continue the arsenauro for at least six months.

The cases which so readily yielded to this antitoxic treatment were apparently of bacterial origin. Arsenauro, by saturating the system, arrested bacterial activity, or killed the germs, or neutralized their toxines. However, only by saturation with the proper medicine—and, by the way, arsenauro is the only powerful alterative neutralizer which can be pushed to an almost incredible dose without doing bodily harm—can such results as are recorded in the foregoing be obtained.

---

***NOTICE.—This JOURNAL and THE INTERNATIONAL JOURNAL OF SURGERY one year for $1.00 cash, to new subscribers. No out-of-town checks accepted unless 10c. is added for cost of cashing.***

---

### CHRONIC DYSPEPSIA SUCCESSFULLY TREATED WITH $H_2O_2$.

The case herewith subjoined is one of interest on account of its typical character, its long standing, and its speedy recovery on the adoption of a rational treatment.

Peter H., aet. 40, Hungarian, farm laborer, applied for treatment at my office on July 1, 1899. He was a strapping fellow, mostly skin and bones, of about 170 pounds weight, and would not have been thought ill except for the prominent dark rings under his eyes, his injected conjunctivæ, and a drawn, hunted expression on his countenance, indicative of past trouble or imminent danger. The history he gave was somewhat as follows:

Six years previously, on his voyage to this country, he suffered from an attack of acute gastritis, attended with retchings of the most violent character. Soon after landing he recovered sufficiently to attend to his work; but he says he has "never been the same man since." In all this long period he has not eaten "a good square meal," nor enjoyed what he has eaten, the burning pain in the epigastrium, after meals, becoming so great occasionally that for fear of its repetition he has gone without food for two or three days at a time. Belching of enormous quantities of gas, too, is common with him soon after eating, thus evidencing the presence of undigested food with its resultant fermentation. The patient states, that in order to get relief he has spent all his wages upon various doctors, specialists, quacks, nostrums, etc., and swears that he is worse to-day than on the day he first landed in this country.

On examination it was found that he was slightly feverish, pulse rapid, tongue flabby and heavily coated, while the teeth and entire cavity of the mouth were covered with a foul-smelling sticky mucus. That the stomach received, in the process of starch digestion, little or no assistance from the salivary glands of the mouth was plainly apparent. In deciding on the mode of treatment it was obvious that lack of the usual amount of gastric secretion must be met by restoring the physiological conditions upon which the secretion depends. In other words, in order to relieve the inflammatory condition of the gastric mucous membrane and restore the function of the peptic glands, antiseptics were required. The patient therefore was furnished with a flask of Ozonized water, made of one part Hydrozone to four parts of water, and directed to wash out his mouth every night and morning, thoroughly cleansing the tongue, teeth and gums of the unhealthly mucous and any pathogenic germs it might contain. To destroy the microbic elements of fermentation in the stomach and dissolve the tenacious mucous there, a mixture of one ounce of Hydrozone with two quarts of sterilized water was made, and half a tumblerful directed to be taken half an hour before meals. Having thus procured a clean surface in the stomach, the patient was advised to take immediately after meals, a dram of Glycozone, diluted in a wineglassful of water, for the purpose of enhancing cellular action and stimulating healthy granulations. Of course he was ordered to select his food with care and eat regularly.

The result of this simple procedure was magical. Although for the first two or three days there was some discomfort after eating, this soon disappeared, and at the end of a fortnight the patient reported that for the first time in six years he was enabled to eat his meals without dread of subsequent distress and eructations of gas. (In the opinion of the writer the fermentation was thus quickly subdued by the active oxidation resulting from the liberation of nascent oxygen.) The treatment was continued in this manner for another month and then gradually abandoned. On September 1st, the patient come to the office, expressed his eternal gratefulness, said that he weighed 185 pounds and believed himself to be completely cured.—*Geo. A. Gilbert, M.D., Danbury, Conn., in New England Medical Monthly, December, 1899.*

---

**This Journal and Atlanta Semi-weekly Journal one year for $1.00 cash in advance. No checks.**

---

### "Panama Fever."

A. O., aged 11, had been subject to irregular attacks of "malaria" for a year or more. Being away from my home during one of these periods another physician was called. To moderate the fever, following a chill, he gave five-grain doses of antipyrine, at that time a comparatively new remedy. This not reducing the fever, even in doses at two or three hour intervals, he applied a Chapman ice bag to spine for two hours. Having to leave town he turned the case over to me, knowing of my return the same afternoon. I found four hours after the removal of the ice bag, a subnormal temperature, it having been 103 degrees six hours before.

The child was in the dorsal decubitas, had complained of no discomfort, was listless, flesh was not cold to the touch, and there was apparently no moisture upon it. There was no tenderness along the spine. Would take food if offered, but expressed no desire for either that or drink. Hot applications externally and various supposedly heating internal medicaments were used with apparently no effect. Two or three visits were made before midnight, but no change was noticed, the patient sleeping quietly, but lightly.

The father, who understood the use of a thermometer and also some of its indications, was given a Hicks certified instrument. Together we took daily and nightly temperatures for the time shown in the record here given. The child, during the days recorded, except during the height of the fever, when there was slight delirium, showed no unusually peculiar symptoms. She would sit up in bed and play with her toys, would answer questions quite as readily as when in good health. The cheeks had a faint tinge of red, though when well she was a fairly rosy child. The discharges from bowels and kidneys showed no marked changes.

One and two hourly thermometer tests were made, but for convenience, I have shown the maximum and minimum for the day. The variations were slight, from record here given.

When at 100 degrees, quinine, with salicylate of soda, was given. The case made a good recovery. No coal-tar, product was used though I had employed antipyrine sparingly in my practice, and after that time not at all—phenacetine taking its place. My remarkable and extremely satisfactory experience with pheno-bromate, in what would have been considered, ten years ago, dangerous doses of any coal-tar derivation, convinces me that great progress has been made in the manufacture of these synthetic products. I have no quarrel with those who use other antipyretics, but it would seem that the use of what appears to be an absolutely harmless, though extremely efficient preparation, would indicate the possession of common sense.

*Mal-de-Mer.*—In ending this article, I must call attention to the fact that pheno-bromate has also proved a valuable friend to "those who go down to the sea in ships," in alleviating and curing seasickness, particularly in those who, coming from the tropics, are in most excellent condition to offer their libations to the deep, and who, while having in every case "weak stomachs," are able, in the words of the volunteer crossing from Tampa to Havana, who, when asked if his stomach was weak, answered: "Yes, sor, but I find I can throw as far as any of them."

I dare not say it is a specific, but I know it relieves the seasickness of those who have sailed with me, and the pyrexia of Panama fever.

WILLIS CUMMINGS, M.D.,
Surgeon Panama R. R. Steamship Line, Pier 57, New York, U. S. A.

### THINGS THEY DO BETTER THAN WE.

There is some sense in an honest man going to law in England; in America, alas, the utmost that a doctor can hope to obtain from an appearance in court against a patient is to escape with his life—and the privilege of paying his own costs. But in England he can actually get justice on the blackmailer. Dr. Davidson, of Gosberton, has just had this pleasure, and deserves the hearty thanks of our entire profession for his high-minded pluck in "carrying the war into Africa." A couple of months ago he received the usual note from a woman who had consulted him a day or two before: "Sir:—Unless you do me some recompense for insulting me the way you did at your surgery, I shall inform some one else about it. It ought to be well worth £10 to you for me to say nothing about it." This has a most familiar sound, but the sequel is a delightful novelty. Dr. Davidson not only rejected her demand but promptly prosecuted her as a blackmailer, and within a month she was found guilty and sentenced to twelve months' imprisonment with hard labor, and most wonderful of all, by a jury. Fancy twelve unterrified American citizens, of the noble and chivalrous type that usually get on our juries, passing such a sentence as this on a woman, and a poor woman at that, for anything she might have done or threatened to do to any "doctor feller." We have known physicians in the Middle West who actually dared not hold any property in the state in which they lived, except a homestead in the name of their wife, for dread of the malpractice and other blackmail suits with which they were threatened, and which would certainly be brought, in full reliance on a sympathetic jury, if any trace of attachable property could be found.—*Jour. A. M. A.*

---

### WHERE WERE THE BLUES AND WHITES?

"Doctor, my husband says black and red spots appear before his eyes every night. What do you advise?"

"I advise that he stop playing poker."—*Chicago Record.*

---

**This Journal and Atlanta Semi-weekly Journal one year for $1.00 cash in advance. No checks.**

### DEAFNESS IN CHILDREN.*

Deafness in children is often allowed to go unnoticed because the child is uncomplaining or the parents careless, and is so often mistaken for dullness and inattention, that the disability becomes fixed and beyond repair before expert skill is solicited.

The most common of the numerous causes of deafness in children is adenoid vegetation in the nasopharynx. Recurrent earache and deafness are symptoms which imperatively demand a thorough exploration of that cavity.

Impaired ventilation and faulty drainage of the nose will be invariably followed by catarrhal inflammation of the middle ear. The importance of such early examination can not be overestimated. The treatment consists in the administration of appropriate constitutional remedies and in the surgical removal of the exciting cause. Incurable deafness in adults can usually be traced to adenoid hypertrophy in childhood. Particular attention is called to recurrent earache and slight deafness as danger-signals given to parents and practitioners, never to be disregarded.

---

**This Journal and Atlanta Semi-weekly Journal one year for $1.00 cash in advance. No checks.**

---

### AN INSTRUMENT OF PRECISION.

"What is a stethoscope and what is it used for?" asked the professor of the class in anatomy.

"The stethoscope," answered the pupil at the pedal extremity of the class, "is a sort of microscope used by a doctor for the purpose of looking into the chest of a patient with his ear."—*Chicago News,*

---

***NOTICE.—This JOURNAL and THE INTERNATIONAL JOURNAL OF SURGERY one year for $1.00 cash, to new subscribers. No out-of-town checks accepted unless 10c. is added for cost of cashing.***

*Abstract of paper read by Dr. Edward F. Parker, of Charleston, S. C., at annual meeting of Tri State Medical Association of the Carolinas and Virginia, February 20, 21, 22, Charleston, S. C.

